# Statistical Assessment of Crosstalk Enrichment between Gene Groups in Biological Networks

**DOI:** 10.1371/journal.pone.0054945

**Published:** 2013-01-23

**Authors:** Theodore McCormack, Oliver Frings, Andrey Alexeyenko, Erik L. L. Sonnhammer

**Affiliations:** 1 Stockholm Bioinformatics Centre, Science for Life Laboratory, Solna, Sweden; 2 Department of Biochemistry and Biophysics, Stockholm University, Stockholm, Sweden; 3 School of Biotechnology, Royal Institute of Technology, Stockholm, Sweden; 4 Swedish eScience Research Center, Stockholm, Sweden; The Centre for Research and Technology, Greece

## Abstract

**Motivation:**

Analyzing groups of functionally coupled genes or proteins in the context of global interaction networks has become an important aspect of bioinformatic investigations. Assessing the statistical significance of crosstalk enrichment between or within groups of genes can be a valuable tool for functional annotation of experimental gene sets.

**Results:**

Here we present CrossTalkZ, a statistical method and software to assess the significance of crosstalk enrichment between pairs of gene or protein groups in large biological networks. We demonstrate that the standard z-score is generally an appropriate and unbiased statistic. We further evaluate the ability of four different methods to reliably recover crosstalk within known biological pathways. We conclude that the methods preserving the second-order topological network properties perform best. Finally, we show how CrossTalkZ can be used to annotate experimental gene sets using known pathway annotations and that its performance at this task is superior to gene enrichment analysis (GEA).

**Availability and Implementation:**

CrossTalkZ (available at http://sonnhammer.sbc.su.se/download/software/CrossTalkZ/) is implemented in C++, easy to use, fast, accepts various input file formats, and produces a number of statistics. These include z-score, p-value, false discovery rate, and a test of normality for the null distributions.

## Introduction

Gene interaction networks are widely used in biological research. Some popular applications include: inference of function for previously unannotated genes [1], extension of GO terms and pathways [2], finding novel disease genes [3], and prioritization of disease gene candidates [4]. However, as always in computational and high-throughput biology, the problems of statistical significance and false discovery need to be properly addressed [5], [6].

When studying statistical properties in biological networks by randomization techniques, a good null model is indispensable. The model should accurately reflect the structure of the original network as well as the conditions that led to its emergence [7]. Various models have been proposed in recent years to model biological networks, in particular PPI networks. Network types include random [8], random geometric [9], and scale free [10]. In all cases, a good null model is a prerequisite for producing reliable predictions that can help to guide experimental studies, while an incorrect model might lead to erroneous conclusions.

**Figure 1:**
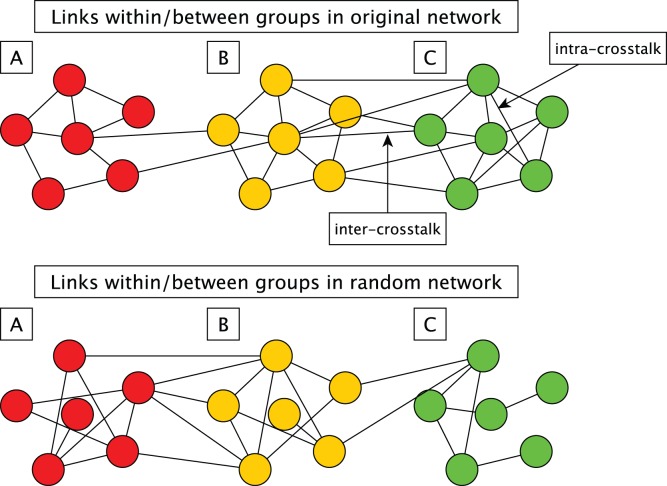
An example of three different cases (enrichment, depletion and no change) of crosstalk. The upper graph shows links in the original network between and within predefined groups (A, B, C) and the lower graph shows an example of the links between groups in a randomized version. Note that only a subset of the networks representing the three groups are shown; all node degrees remain constant after randomization. In this example, groups A and B have fewer links between them than in the random case (inter-group depletion) while B and C have more links (inter-group enrichment). Also seen in this example is that group B and C have intra-group enrichment while group A has the same number of links in the original as in the random network (no enrichment or depletion).

We define crosstalk enrichment in biological networks as the extent of connectivity between (inter) or within (intra) biological groups (see Figure 1). An experimental gene set, a known functional module, or a pathway are all examples of biological groupings. To draw statistically sound conclusions about crosstalk enrichment or depletion, a probabilistic estimate is crucial.

A probabilistic estimate is produced by comparing an observation to a reference. The reference is determined by the null model hypothesis, which must express a distribution of the number of observations expected by chance. In network analysis, an observation is typically the number of links found within a certain network structure, e.g. inside of a network module or between two modules. Given the scale-free property of most biological networks, it does not make sense to compare the properties of network hubs to those of sparsely connected genes without such a reference. For instance, a hub gene may have a few links to genes of a certain module simply by chance, whereas for a gene with a modest number of connections, the same number of links would indicate an important biological pattern. If a null distribution is available, then both the expected number of links and the respective variance can be estimated.

In simple cases, the number of links expected by chance can be estimated analytically for scale-free networks, for instance, from the hyper-geometric distribution [11]. However, analytical methods are not feasible for more complex cases, such as when gene groups share members, which is likely to exaggerate the number of links between them. For example, when Li, Y. et al. [12] built a network of pathways by computing the crosstalk between pathway pairs using the Fisher Exact test, they had to limit their analysis to only non-overlapping pathway pairs.

An alternative way to estimate the null model distribution, or the links expected by chance, is to perform numerical simulations. Huttenhower et al., analyzed crosstalk between gene groups within biological processes by estimating z-scores using a null model for group size combinations [13]. Because this approach is based on regression across gene group sizes within each process, it is not flexible enough for application to any pair of gene groups.

Early work on comparing the real network to its randomized version in the context of biological networks was done by Maslov and Sneppen [14]. They applied the classical switch randomization algorithm [15], which randomly rewires the original network such that the connectivity, or degree, of every node is preserved. This method was also used by Milo et al. to find small network motifs occurring significantly more often in biological networks than expected in corresponding randomized networks [16]. Kuchaiev et al. used a geometric graph model for assessing confidence levels of known interactions in PPI networks and for predicting novel ones [17]. Although the literature on network randomization theory is large, this has so far not been applied to estimate the statistical significance of the connectivity within or between gene groups in gene networks.

For such crosstalk analysis, we found that the distribution of the expected number of links approaches normal if the network pattern of interest has sufficiently many links in a series of random instances. In this case, the standard z-score can be computed by dividing the difference between observed and expected number of links with the expected links’ standard deviation. Z-scores can trivially be transformed into p-values, which are useful since they can readily be adjusted for multiple testing. This statistical approach has been applied to analyze crosstalk between co-mutated genes in cancers and between candidate disease genes [4], [18], [19].

Maslov and Sneppen also discussed the existence of second-order non-random patterns [14]. For instance, two networks with identical node degree distributions can have different patterns of connectivity between hubs and non-hubs. Network assortativity describes the preference of nodes to connect to nodes of similar or dissimilar node degree [20]. In biological networks, Newman detected negative assortativity, i.e. the tendency of hubs to avoid each other and instead preferentially attach to low degree nodes. An alternative to assortativity is to sum the product of connected nodes degrees for all links in the network, termed the s-metric [21]. If either of these metrics change during randomization, then the second-order structure of the network has been altered. In the case of crosstalk analysis, it is desirable to ensure that they stay as constant as possible when generating null models [22].

When analyzing high-throughput data, one of the key interests is to shed light on the underlying biology. The most common strategy to functionally annotate gene groups is to perform gene-set enrichment analysis (GEA) [23]–[25], i.e. to look for functional annotations that are statistically over-represented within a group of genes. Despite being popular, GEA approaches are limited in that they entirely depend on the completeness and quality of collected functional annotations. At present, many genes still lack functional annotations, for example in form of Gene Ontology (GO) terms [26] or KEGG pathways [27]. GEA methods further fail to identify pathway annotations that are strongly linked to a gene group, but only have a few shared genes.

Here we compare the suitability of four different network randomization algorithms for statistical crosstalk enrichment analysis [15], [28]. The methods are evaluated with respect to their true and false positive rates, conservation of topological properties, the null model distribution quality, and the computational speed under different conditions. Furthermore, we show that crosstalk enrichment analysis can be a valuable tool for functional annotation of gene groups. We compare crosstalk enrichment analysis GEA and demonstrate that its performance is superior to GEA when annotating experimental gene groups from MsigDB [24] using know pathway annotations from KEGG [27].

We also present CrossTalkZ, a software package that implements the four randomization techniques and quantifies the connectivity between gene groups of interest. The package is freely available and includes several statistical tests to allow the user to draw solid statistically supported conclusions.

## Results

In undirected networks, crosstalk between (inter) or within (intra) gene groups is defined as the extent of connectivity between the genes in the groups (see Figure 1). To assess the significance of crosstalk enrichment, we compare the observed connectivity with the connectivity expected from a null model distribution. The expected connectivity between groups is obtained by tallying the links between genes of the two groups after randomizing the network multiple times. We first show that if the distribution of expected links is normal, the standard z-score is an appropriate and unbiased statistic to estimate the significance of crosstalk enrichment and that other statistics such as a p-value and false discovery rate (FDR) [29] can readily be calculated. We then compare four different network randomization algorithms with respect to their suitability for crosstalk analysis. Finally, we exemplify how crosstalk enrichment analysis can be used to annotate experimentally derived gene groups and that it performs favorably compared to gene set enrichment analysis (GEA).

### Network Randomization Methods

There are many different ways to randomize networks. We pose a minimum requirement that the degree distribution is preserved. In many cases it is also important to preserve second-order topological patterns of the original network to best represent the system under study. We tested four different randomization methods, each with varying degrees of topological conservation, for their ability to discover crosstalk. The methods are referred to as link permutation (LP), node permutation (NP), link assignment (LA), and link assignment+second-order conservation (LA+S). The LP method, randomly swaps links in a network. NP permutes node labels between nodes that have similar degree. LA implements a variant of the matching algorithm [15] or configuration model [28] by adding an extra step to resolve the problem of self-links or multiple links between nodes. It starts with an unconnected network and randomly adds links between nodes until the original node degrees are recovered. LA+S is a variant of LA that also preserves the second-order topology. All of the methods preserve the network degree sequence (distribution) of the original network.

### Estimating the Significance of Crosstalk Enrichment

The estimation of the significance of crosstalk enrichment, i.e. the extent to which the connectivity between or within functional groups exceeds what is expected by chance, is a crucial aspect of crosstalk analysis. The converse of enrichment is depletion, and corresponds to observing less connectivity than expected by chance.

There are two common alternatives for significance estimation. The first is to use a permutation test where a p-value can be calculated as the fraction of null cases (i.e. observations in randomized network instances) that contained more links than observed in the real network. An alternative is to use standard z-scores, which for the normal distribution, is the number of standard deviations away from the mean that an observation point lies. The z-score can readily be transformed into a p-value. One of the crucial differences between the two methods is that the z-score converges after relatively few randomizations, while a high precision indirect p-value estimation using a permutation test requires a very large number of permutations to converge. Because, the full permutation test is computationally expensive, we opted for the z-score alternative. Another advantage of using the z-score is that it is easily interpreted as crosstalk enrichment or depletion, where positive values indicate enrichment and negative values indicate depletion.

**Figure 2:**
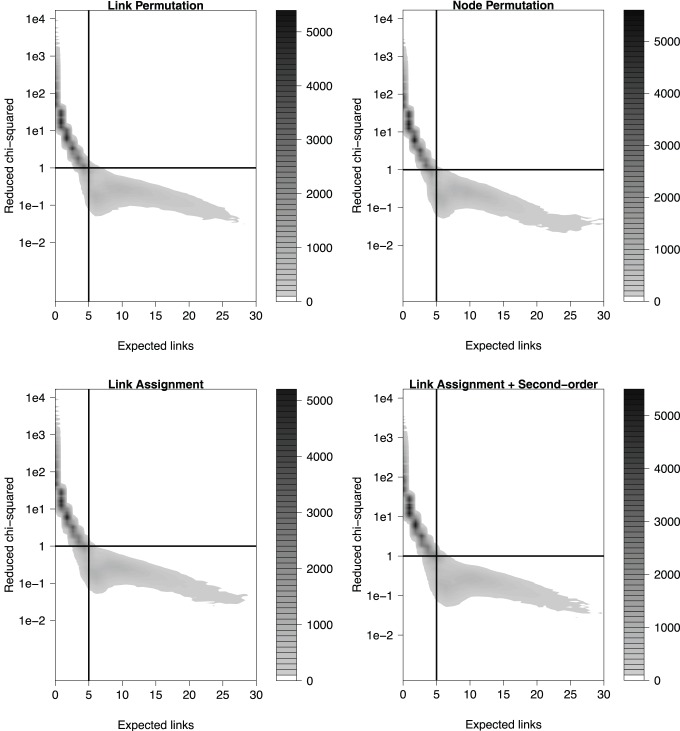
Normality test of the null model distribution. Values of the reduced chi-squared test of normality are plotted against the expected number of links for all four methods using randomized KEGG pathways. The normality criterion is generally only violated for low numbers of expected links. The horizontal line represents the threshold used for normality (chi-squared = 1), and the vertical line is at expected number of links = 5. The number of points is indicated by the density, lighter colors are lower density.

In order for the z-score and p-value to be meaningful, the null model distribution needs to fulfill the normality criterion. To assure that the assumption of normality is fulfilled, a reduced chi-squared statistic is calculated. We generated 100 sets of randomized KEGG pathways to test for this. Every node in each pathway was replaced with a random node from the entire FunCoup network of similar degree. In this way, each pathway contains new random genes while approximately conserving its original degree sequence. Figure 2 shows the reduced chi-squared statistic versus the number of expected links for all four methods for this test. We found that the normality assumption is generally only violated if the number of expected links is small. For the set of randomized KEGG pathways we found for example that when averaged over all four methods, only 4.7±1.5% of the cases had expected number of links below 5 and fit a normal distribution, i.e. reduced chi-squared < = 1 [30]. On the other hand, for the cases with expected links above 5, 97.6±0.8% had reduced chi-squared < = 1. One should note, however, that reduced chi-squared = 1 can potentially be an overly stringent criterion if the expected number of links is small, leading to false negatives.

**Figure 3:**
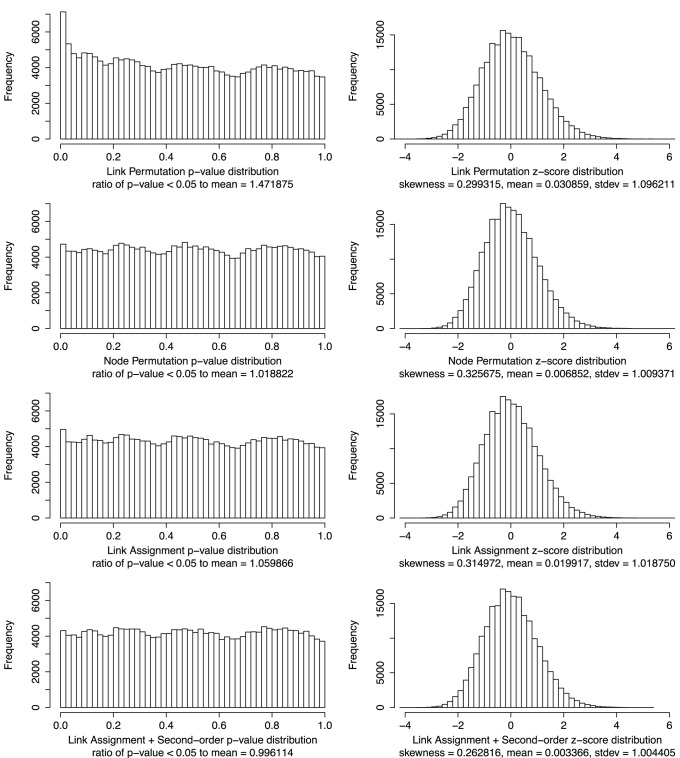
Comparison of the p-value and z-score distributions for all four methods. The analysis were performed on a set of randomized KEGG pathways. All results were filtered for reduced chi-squared < = 1.0. According to the central limit theorem the distribution of z-scores between random groups should be standard normal and therefore give a uniform distribution of p-values. For each p-value distribution we report the ratio between the number of p-values <0.05 and the average number in equally sized bins with p-value > = 0.05. An unbiased distribution should give a ratio of 1. For each z-score distribution we report skewness, mean and standard deviation. For each method 150 rando­mizations were performed.

For the analysis to be unbiased, the central limit theorem suggests that the distribution of z-scores between random groups should be standard normal and therefore give a uniform distribution of p-values. For the randomized KEGG pathways these distributions are shown in Figure 3, along with some quality metrics. When considering only test cases that passed the expected links normality criterion, all four methods generated approximately standard normal z-score distributions. However, LA+S was clearly the best and LP the worst when looking at skewness, mean, standard deviation, and p-value bias. A bias in the p-value distribution can be seen by looking at the ratio of the number of points with p-value in the range [0, 0.05] to the average number of points in equally sized bins for p-value > = 0.05. The least biased method was LA+S with a ratio of 0.996, while LP was the worst with almost 50% more significant p-values than the mean in insignificant bins.

### Preservation of Second-order Network Topology

We tested to which degree the different methods preserve the topology of the original network by calculating two different measures: assortativity [20] and the s-metric [21]. The network assortativity is the correlation between the degrees of the two nodes connected by a link, while the s-metric is simply the sum of the product of node degrees that are connected by a link. The human FunCoup network had an assortativity of r = 0.2, indicating that high degree nodes are more frequently connected to high degree nodes than to low degree nodes. We observed the same trend for the human STRING [31] network (cutoff >0.5, r = 0.32). This is opposite to what Newman reported for biological networks (r = −0.15) [20]. Independently, Maslov and Sneppen found biological networks to be disassortative (r <0), i.e. that high degree nodes have a preference to connect to low degree nodes [14]. However, the networks used in their studies were relatively small yeast networks, purely based on physical protein interactions.

The average assortativity, s-metric ratio, and percent identity for each of the four methods are shown in Table 1. By percent identity we mean the fraction of links shared between the original and the randomized networks. A method with higher percent identity can be considered more conservative than one with a lower percent identity. As expected, NP preserved the assortativity and s-metric of the original network, simply because it did not change the network topology. The other methods altered these metrics to varying degrees. The effect for the LA+S method was marginal, as it aimed to connect nodes that have same degree as in the original network. For the less conservative methods LP and LA, the assortativity turned negative and the s-metric was substantially lower than for NP and LA+S. Depending on the null hypotheses tested, conclusions based on the expected number of links might be biased if the network randomization does not account for second-order topological features of the original network. Both LA+S and NP should generate a null distribution that preserves the second-order topology of the original network and are therefore preferable for crosstalk analysis.

**Table 1:** Shown are the means and standard deviations for one primary (column two) and two secondary (column three and four) topology conservation metrics for the four different network randomization methods.

Method	Percent identity of links betweenrandomized and original	Randomized assortativity (original r = 0.20)	s-metric ratio (randomized/original)
LP	7.38±0.04	−0.09±0.00	0.80±0.00
NP	12.12±0.11	0.20±0.00	1.00±0.00
LA	10.47±0.21	−0.15±0.02	0.76±0.01
LA+S	14.79±0.07	0.14±0.01	0.96±0.00

The average percent identity is the fraction of links the randomized network had in common with the original network. The assortativity represents the correlation between degrees of connected nodes. A positive assortativity indicates that nodes tend to be connected to nodes of similar degree, while a negative assortativity indicates that they tend to connect to nodes with different degrees. The s-metric is another link degree correlation measure; the ratios shown are the randomized network’s s-metric divided by the original network’s s-metric. All results shown were generated from 50 iterations for each method using the human FunCoup network.

### Estimation of False Positive Rate

To analyze the false positive rate of the four algorithms we used the same dataset of sets of randomized KEGG pathways previously used for analyzing distribution properties (Figure 2, 3), as a source of crosstalk statistics between pathways that should not have crosstalk. In a first test we used a simple cutoff such that a crosstalk enrichment measurement was considered a false positive if its p-value <0.05 and it had a reduced chi-squared value < = 1.0. The least conservative method, LP, gave the highest fraction, 4.7%. LA+S gave the lowest fraction, 3.2%, slightly lower than NP and LA, at 3.5% and 3.6%, respectively.

**Figure 4:**
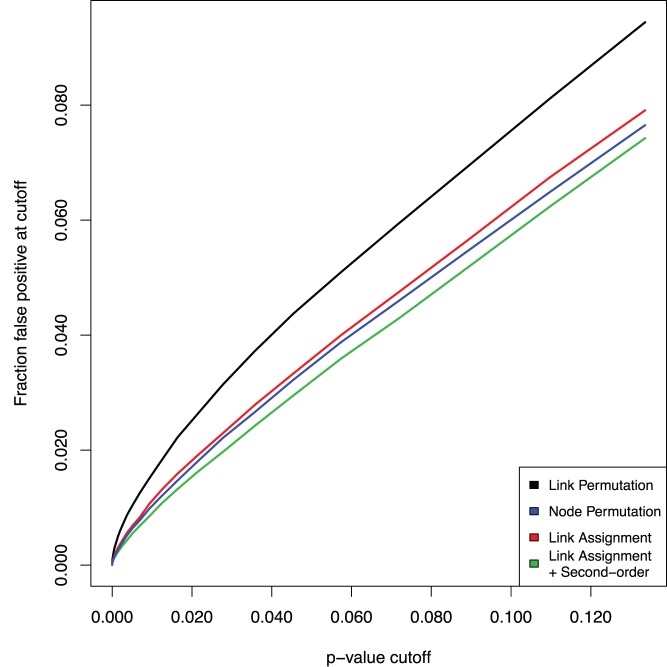
Comparison of the false positive rate for the four different methods. A result was considered false positive if it passed the normality criterion (reduced chi-squared < = 1) and had a p-value below a certain cutoff. Shown is the false positive rate as a function of the p-value. The test was performed using randomized KEGG pathways mapped to the FunCoup network.

In a second, more comprehensive test, we analyzed the false positive rate for each method across a range of p-values (Figure 4). This also shows that the LP method is clearly less conservative than the other methods, and that the LA+S method is most conservative.

### Estimation of False Negative Rate

Hundreds of signaling, metabolic and other functional gene groups are known from the literature and found curated in databases, such as GO [26] or KEGG [27]. Li et al. demonstrated that GO terms and pathways (HumanCyc and BioCarta) are often enriched in internal network connections [12]. Based on the assumption that pathways are functionally coherent groups, we analysed intra-crosstalk within KEGG pathways mapped to FunCoup as a positive control, to estimate the false negative rate of the methods.

**Figure 5:**
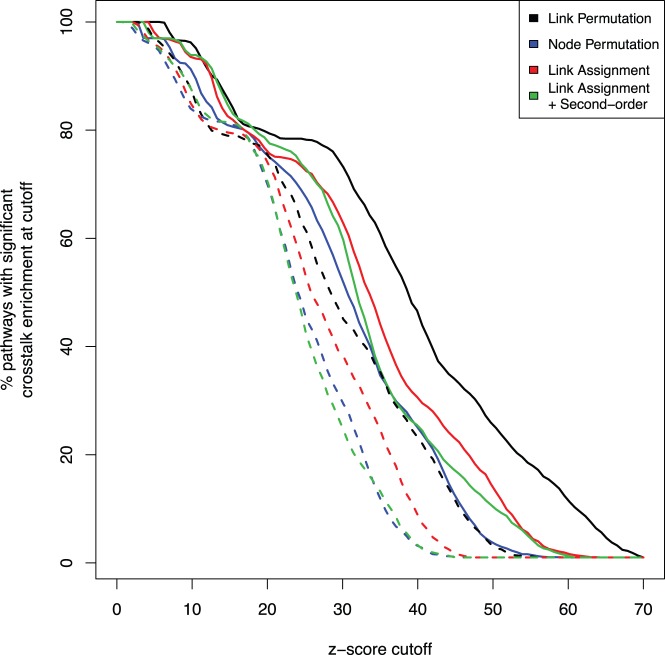
Comparison of the false negative rate for the four different methods. Shown is the percentage of correctly recovered pathways (i.e. 1– false negative rate) as a function of the z-score. Solid lines represent internal crosstalk enrichment detection rates while dashed lines represent inter-crosstalk enrichment detection rates for KEGG pathways that were split into random halves. The test was performed using KEGG pathways mapped to the FunCoup network.

At FDR <0.05 and reduced chi-squared < = 1.0, all four methods found 100% of the pathways to be significant for this test. However, at higher stringency the differences between the methods are more apparent. To show this, we plotted the fraction of significant internal crosstalk as a function of the z-score (Figure 5, solid curves). We used the z-score here to maximize the range of sensitivity. Consistent with the fact that the LP method previously showed the highest false positive rate (Figure 4), it also showed the lowest false negative rate here (i.e. the highest recovery rate). Although the LA+S method previously showed the lowest false positive rate, it did not have the highest false negative rate. In fact, around a z-score cutoff of 15 it is the best method. Overall LA+S thus appears to be the best compromise between false and negative rates.

A variation of this test involves splitting each pathway approximately in half resulting in two pathways. In this test we look at the ability of the methods to re-discover the crosstalk between the pathway halves. Since we observed strong intra-crosstalk enrichment in the original KEGG pathways, we expect that splitting these pathways in random halves should result in strong inter-crosstalk enrichment between the halves. At FDR <0.05 and reduced chi-squared < = 1.0, all methods recovered at least 99.1% of the pathways. The dotted lines in Figure 5 represent the results of the split pathway test. Again, the LP method showed the lowest false negative rate here, while the LA+S and NP methods performed about equally, showing the highest false negative rates.

The main outcome of both the negative and positive controls is that LP performs strikingly less conservative than the other methods as it estimates higher z-scores. The other three methods perform similarly, but the two methods that preserve second-order topology well, LA+S and NP are more conservative in most tests.

### Pathway Analysis

An important goal of analyzing high-throughput data, such as lists of differentially expressed genes, is to study the underlying biology. A commonly applied approach is to perform gene enrichment analysis (GEA), i.e. to look for functional annotations that are statistically over-represented within a group of genes [32]. While GEA approaches are popular and easy to implement, they are limited in certain ways [23]. They entirely depend on the completeness and quality of pathway annotations, both of which are low at present. In contrast, crosstalk enrichment analysis by CrossTalkZ looks for enrichment in network connections between two groups of genes. Because it employs the network, it can detect pathways that are strongly associated to a gene group, even when GEA can not detect this because of few shared genes. It may also detect associations to relevant up- or downstream pathways if the crosstalk is sufficiently strong.

To compare these approaches, we applied both CrossTalkZ and GEA to search for associations between 2392 experimental gene groups from MsigDB [24] and 236 KEGG pathways. GEA found 3370 unique, i.e. only found by GEA, gene group to pathway associations that were significant. CrossTalkZ using the FunCoup network found 49225 unique significant associations while 9707 significant associations were found by both methods. In other words, CrossTalkZ provides a very substantial increase in pathway annotations compared to GEA, yet finds most GEA annotations. To illustrate the usefulness of CrossTalkZ, we briefly discuss two examples from this screen.

Setlur et al. [33] presented a gene signature of 63 genes up-regulated in prostate cancer. The GEA approach failed to identify any significant KEGG pathway associations (see Table 2). In contrast, CrossTalkZ identified 9 significant KEGG pathway associations that reflect the functional dynamics of the signature. We found significant associations to pathways “Prostate cancer” and “Pathways in cancer”. We further found strong links to the TGF-beta signaling pathway which plays a well-studied role in tumorigenesis and cancer progression [34], as well as the Notch signaling pathway which has been linked more recently to prostate cancer as one of the key regulators of prostate cancer progression [35].

**Table 2:** KEGG pathway associations to a 63 gene signature of genes up-regulated in prostate cancer.

KEEG pathway associations with prostate cancer signature by Setlur et al.				
	Z-score CrossTalkZ	ChiSqr CrossTalkZ	FDR CrossTalkZ	FDR GEA
TGF-beta signaling pathway	4.58	0.02	5.31E−06	0.19
Basal transcription factors	4.55	0.05	5.95E−06	1.00
Notch signaling pathway	4.01	0.32	7.20E−05	0.50
Ubiquitin mediated proteolysis	3.26	0.01	1.41E−03	0.85
Pathways in cancer	3.11	0.03	2.34E−03	1.00
Prostate cancer	2.98	0.10	3.71E−03	1.00
Proteasome	2.59	0.04	1.30E−02	0.49
Cell cycle	2.44	0.02	2.06E−02	0.26
Bladder cancer	2.26	0.36	3.46E−02	1.00

Shown are the z-score, the reduced chi-squared, and the FDR for CrossTalkZ as well as the FDR for the GEA approach. CrossTalkZ identified 9 significant pathway associations while GEA failed to identify any significant pathway associations.

A signature of 389 genes up-regulated in a sub-cluster of bladder cancers dominated by G2 tumors was presented by Lindgren et al. [36]. CrossTalkZ identified 81 significant pathway associations of which 55 were not found by the GEA approach. Among the pathways identified by both methods were various pathways related to cellular functions as cell migration, differentiation, proliferation, and apoptosis. Pathway associations only found by CrossTalkZ included: the KEGG bladder cancer pathway, TGF-beta signaling pathway, Wnt signaling pathway, ErbB signaling pathway as well as many other cancer-related pathways (see Table S1).

### Software Performance

**Figure 6:**
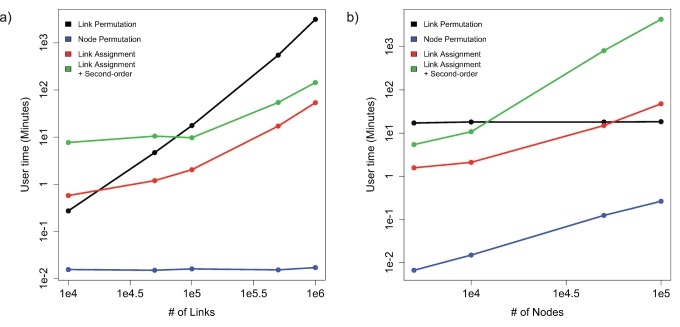
Software performance for 150 randomizations for each of the four methods. a) Computational performance with increasing number of links and constant number of nodes. The node permutation method is independent of the number of links and only depends on the number of nodes. b) Computational performance with increasing number of nodes and constant number of links. The link permutation method is independent of the number of nodes and only depends on the number of links.

We tested the performance of each of the four methods on random scale free networks that were obtained using the Barabási–Albert scale free model [10]. Figure 6 shows the performance of the methods for 150 randomizations when either a) keeping the number of nodes constant and varying the number of links or b) keeping the number of links constant and varying the number of nodes. The node permutation method is the fastest in all test cases and its speed depends only on the number of nodes in the network. Conversely, the link permutation method depends only on the number of links, but is orders of magnitude slower on the test set. The link assignment methods have a more complex performance curves, but still randomize a network of 10̂4 nodes and 10̂6 links in approximately 10 seconds. All benchmarks were conducted on a 2 GHz processor with 4GB of memory.

## Discussion

We created CrossTalkZ, a method and software to assess the significance of crosstalk, which we define as the extent of connectivity between or within gene groups in biological networks. We first showed that the significance of the crosstalk enrichment can be assessed with the standard z-score, which compares the number of links observed between gene groups in the original network to the number of expected links. The expected number of links under the null model is estimated by generating a series of randomized instances of the original network. Second, we compared four different randomization methods with regard to their suitability for crosstalk enrichment analysis. Third, we showed that crosstalk analysis performed favorably to gene-set enrichment analysis (GEA) when annotating experimental gene groups from MsigDB.

CrossTalkZ implements four network randomization algorithms. The algorithms conserve the scale-free topology (degree distribution) of the original network and differ mostly by the extent to which they conserve second-order topological properties. Link permutation attempts to swap *all* links in the original network and therefore generates random networks that have the least links in common with the original network. As a result, it is potentially underestimating the number of links between groups expected by chance, giving overall higher fractions of significant crosstalk enrichment. Link assignment provides random networks that have links between nodes drawn uniformly from the whole network, a result of which is disassortative mixing (high degree nodes prefer low degree nodes). The more conservative methods LA+S and NP have restricted sets of links or nodes, respectively, to choose from when randomizing and therefore potentially over estimate the number of links between groups expected by chance, resulting in lower fractions of significant crosstalk enrichment.

We compared the algorithms with respect to their ability to produce high-quality random networks in order to provide unbiased null distributions for the estimation of the expected number of links. Overall, we found that the methods LA+S and NP maximally preserved the topological properties of the original network and gave the lowest fraction of both true and false positives, as expected. Interestingly, the LA+S method minimized the fraction of false positives while preserving second-order properties to a similar degree as NP. Furthermore, it produces the least biased z-score and p-value distributions of all the methods and is therefore the default method in the CrossTalkZ software package. Conversely, the least conservative methods LP and LA resulted in higher fractions of both true and false positives and produced more biased p-value distributions.

Gene enrichment analysis is often applied for pathway annotation of gene groups, for example, sets of differentially expressed genes. However, it often fails because of the incomplete state of current pathway databases. Methods that employ connections in a comprehensive interaction network are more likely to identify pathway associations. We exemplified how CrossTalkZ using the FunCoup network can be a valuable addition to classical gene enrichment analysis and that it can uniquely find many relevant pathway associations.

Since our statistics rely on a normal distribution of the expected number of links, our method gives completely unbiased p-value estimates only on groups with a sufficient number of expected links in the randomized networks. In general, the reliability of the statistics can always be assessed by the reduced chi-squared statistic, which conveys the degree of deviation from normality. Potentially (in a future software version), this bottleneck could be circumvented by directly estimating the false positive rate for a given case by a permutation test.

## Methods

### Network Randomization Algorithms

#### Link permutation (LP)

This method swaps links between nodes while conserving their node degree. In order to swap links, two links are chosen randomly from the network. Then, if the links do not share common nodes and if swapping the links forms two new links in the network, the links are swapped. This process is repeated until all the links in the original network have been swapped or no additional swapping can occur. Since swapping links leaves the degree of each node involved unchanged, the randomized network conserves degree sequence.

#### Node permutation (NP)

In this method, network topology is completely conserved because only the nodes are permuted randomly. In our implementation, a given node may only swap with a node that belongs to the same connectivity bin, so as to approximately conserve its connectivity. Bin borders were dynamically established using a natural log scale where a node with degree *d* falls into bin B(*d*) = round(ln(*d*)+1).

#### Link assignment (LA)

This method begins by removing all the links from the network while keeping track of each node’s degree. Links are assigned between randomly chosen nodes until the original degrees are restored *or* no more nodes satisfy the connection constraints. These connection constraints are as follows: no self-loops or multiple edges are allowed, and the degree of both nodes must be less than or equal to their respective original degree after link assignment. Once a node recovers its original degree, it is removed from the set of available nodes. Since it is possible that some nodes will not recover their original degree due to connection constraints, a final test and degree fix by swapping procedure is applied to those nodes (see Text S1).

#### Link assignment+second-order conservation (LA+S)

The “neighbor degree sequence” describes the association between a given node’s degree to the degree of nodes connected to it. With this in mind, we introduce a variation of our link assignment method termed link assignment+second-order conservation. This method uses the same basic approach as LA, but for a given node, instead of choosing any node available in the network to connect to, the available nodes are restricted to a set of nodes that fall into the same log connectivity-bin as nodes it was connected to in the original network. After randomization, the degree sequence is conserved and each node has a similar neighbor degree sequence as in the original network.

### Statistics

After generating a normally distributed null model, a standard z-score can be calculated as follows: *Z_ij_ = (Nobs_ij_−Nexp_ij_)/SD_ij_* where *Nexp_ij_* is the mean number of links between groups i and j found after *N* randomizations of the network, *Nobs_ij_* is the number of links found between groups i and j in the original network, and *SD_ij_* is the standard deviation of the number of links between the two groups after *N* randomizations. The z-score is further transformed into a p-value and adjusted for multiple testing using the false discovery rate (FDR) with the procedure of Benjamini and Hochberg [29]. When applying CrossTalkZ in research one should always use FDR as a criterion for the significance of cross-talk. However, for illustrative purposes we use p-values or z-scores in Figures 4 and 5 as it makes differences between the methods more apparent. P-value adjustment is conducted separately for inter- and intra- crosstalk as they are considered as two independent tests. As a measure of how well the distribution of expected links for each pair of gene groups fits a normal distribution, a *reduced* chi-squared statistic is calculated [30]. The reduced chi-squared normalizes for the number of degrees of freedom in a statistical calculation, which in this implementation is *d = N−c,* with *N* the same as above and *c = 3* the number of constraints (mean, standard deviation, and *N*). Taylor suggested that for reduced chi-squared of order one or less the observed distribution fits the theoretical distribution well [30]. Therefore, in our analysis we only used results with reduced chi-squared < = 1 to ensure the z-scores calculated are valid.

### Interaction Network

The FunCoup database provides global interaction networks for a variety of species and combines different types of evidence: protein-protein interactions, mRNA co-expression, sub-cellular co-localization, phylogenetic profile similarity, co-targeting by either miRNA or transcription factors, protein co-expression, and domain-domain interactions. It further transfers evidence from other eukaryotic species via orthologs. For this study we used the human FunCoup v1.1 network. All links with a confidence cutoff of 0.5 or higher were included. We earlier demonstrated that this network possesses the scale-free property [18], which is typically associated with biological networks. The whole network included 10885 unique genes with 230589 links between them. The highest and most rare node degree was 957 while the lowest and most frequent node degree was 1. From this point, all references to the FunCoup network will be referring to the network described here.

### Pathway Data

We collected all human pathway annotations from the KEGG database (as of February 2010) [27]. All pathways containing less than 10 genes present in the interaction network were excluded, since they are likely to have a low number of expected links between them and would therefore not fulfill the normality criterion. In total we included 66 metabolic and 33 signaling human KEGG pathways, covering 2004 unique genes. Out of the original 2650 unique KEGG genes, 587 were not represented in the interaction network. From this point, all references to KEGG pathways will be referring to the pathway set described here.

### Random Pathways

To assess the false positive rate of the different randomization methods, we created random pathways that resemble the original KEGG pathways in both the number of genes as well as the degree distribution. For each original pathway we generated 100 random pathway instances. To preserve the degree distribution of the original pathway, each gene was replaced with a gene from the network that had similar connectivity; i.e. the node degree had to fall into the same log connectivity-bin. Swapping of genes was further restricted by two conditions: the new gene could not be in the original pathway and no duplicate genes were allowed in a pathway. Thus, each random pathway instance contained the same number of genes randomly drawn from the full set of network nodes while approximately preserving its original degree distribution.

### Pathway Analysis

We performed a large scale screen between gene signatures from MsigDB [24] and KEGG pathways. In total we considered 2392 gene signatures from the category C2: chemical and genetic perturbations. The KEGG pathway set was comprised of 236 metabolic, signaling, and disease pathways from KEGG. Significance of signature-pathway associations was assessed by CrossTalkZ using the FunCoup network as well as gene enrichment analysis.

GEA was performed by calculating the probability of the overlap between gene signatures from MsigDB and KEGG pathways using the hypergeometric probability distribution: P(X = k) = C(m, k) * C(N−m, n−k)/C(N, n). Where *C(x)* is the binomial coefficient, *N* is the total number of unique genes found both in MsigDB and KEGG, *m* is the KEGG pathway size, *n* is the gene signature size, and *k* is the number of successes i.e. the number signature genes that are part of a respective KEGG pathway. All p-values were adjusted for multiple testing using the false discovery rate (FDR) with the procedure of Benjamini and Hochberg [29].

A pathway-signature association was considered significant if it had an FDR <0.05 and in the case of crosstalk analysis also had a reduced chi-squared <1 and a z-score >0.

### Implementation

All four methods have been implemented in a single package called CrossTalkZ. The package was written in C++ and used the Libxml2 (xmlsoft.org) and Boost graph libraries (boost.org). CrossTalkZ requires two files as input: a network file and one or two group file(s). If one group file is given, statistics for all pairwise permutations of groups are calculated. If two group files are given, statistics for the first set of groups versus the second set of groups are calculated. For more details see Text S2.

When counting links between groups in either the random or the original network, the question of how to treat links between groups that have common genes must be addressed. Therefore, two different link counting modes are implemented in CrossTalkZ. For inter-crosstalk, the default link counting mode does not tally a link that has *either* of its nodes in both of the groups. The alternate mode is to not tally a link that has *both* of its nodes in both groups. Intra-crosstalk links are always tallied.

## Supporting Information

Table S1 **Significant KEGG pathway associations to a 389 gene signature up-regulated in bladder cancer.**
(PDF)

Text S1 **Pseudocode for randomization algorithm.**
(PDF)

Text S2 **Implementation Details.**
(PDF)
