# Supplementary material for: Statistical Assessment of Crosstalk Enrichment between Gene Groups in Biological Networks
Source: PLoS One. 2013 Jan 23;8(1):e54945. doi: 10.1371/journal.pone.0054945 (PMC3553069; doi:10.1371/journal.pone.0054945)
Supplement: Table S1 — Significant KEGG pathway associations to a 389 gene signature up-regulated in bladder cancer. (PDF) [file pone.0054945.s001.pdf]

Table S1: Significant KEGG pathway associations to a 389 gene signature up-regulated in bladder cancer.

KEGG	FDR-CrossTalkZ	FDR-GEA
B cell receptor signaling pathway	0.00E+00	2.53E-04
Cell adhesion molecules (CAMs)	0.00E+00	2.93E-10
Chemokine signaling pathway	0.00E+00	5.75E-06
ECM-receptor interaction	0.00E+00	1.57E-07
Fc epsilon RI signaling pathway	0.00E+00	1.27E-01
Fc gamma R-mediated phagocytosis	0.00E+00	1.08E-02
Focal adhesion	0.00E+00	5.11E-12
Jak-STAT signaling pathway	0.00E+00	9.83E-04
Leukocyte transendothelial migration	0.00E+00	3.70E-10
MAPK signaling pathway	0.00E+00	1.55E-03
Natural killer cell mediated cytotoxicity	0.00E+00	4.02E-04
Pathways in cancer	0.00E+00	1.66E-03
Regulation of actin cytoskeleton	0.00E+00	4.42E-05
T cell receptor signaling pathway	0.00E+00	8.72E-06
Bacterial invasion of epithelial cells	1.41E-45	2.64E-01
ErbB signaling pathway	1.41E-45	4.68E-01
VEGF signaling pathway	4.37E-43	1.10E-01
Neurotrophin signaling pathway	8.41E-42	5.05E-03
Leishmaniasis	1.85E-40	4.37E-01
GnRH signaling pathway	4.65E-40	2.00E-01
Adherens junction	1.12E-38	1.55E-01
Cytokine-cytokine receptor interaction	7.40E-38	1.03E-04
Hematopoietic cell lineage	4.29E-37	1.55E-01
Glioma	4.59E-37	1.10E-01
Gap junction	2.51E-35	1.80E-01
Axon guidance	4.00E-35	4.59E-04
Prostate cancer	9.70E-35	6.62E-02
Acute myeloid leukemia	9.72E-34	2.93E-01
Viral myocarditis	1.05E-32	1.24E-03
Toll-like receptor signaling pathway	1.41E-32	4.57E-01
Pancreatic cancer	2.06E-31	1.60E-01
Renal cell carcinoma	4.11E-30	7.25E-01
Non-small cell lung cancer	5.96E-29	9.96E-01
Colorectal cancer	1.66E-27	3.24E-03
Intestinal immune network for IgA production	6.78E-27	4.80E-02
Small cell lung cancer	1.16E-25	4.14E-03
Dorso-ventral axis formation	2.79E-25	9.96E-01
Insulin signaling pathway	4.52E-24	8.36E-02
Chronic myeloid leukemia	6.97E-24	4.00E-01
Tight junction	3.34E-23	5.94E-01
Long-term potentiation	3.75E-23	9.96E-01

Chagas disease (American trypanosomiasis)	7.59E-22	4.29E-01
Vascular smooth muscle contraction	1.42E-21	4.07E-02
Endometrial cancer	4.16E-21	5.84E-01
Epithelial cell signaling in Helicobacter pylori infection	6.56E-20	3.21E-01
Pathogenic Escherichia coli infection	3.90E-18	9.29E-01
Melanoma	5.30E-17	3.41E-01
Adipocytokine signaling pathway	6.30E-16	1.37E-01
Apoptosis	4.50E-15	6.37E-02
Calcium signaling pathway	6.62E-15	6.57E-02
Antigen processing and presentation	2.95E-14	1.15E-04
Complement and coagulation cascades	2.95E-11	1.07E-03
Cytosolic DNA-sensing pathway	6.11E-11	7.52E-01
Hypertrophic cardiomyopathy (HCM)	1.92E-10	3.15E-01
Type II diabetes mellitus	1.95E-10	2.11E-01
RIG-I-like receptor signaling pathway	1.99E-10	3.95E-01
TGF-beta signaling pathway	2.38E-10	1.80E-01
Bladder cancer	3.37E-10	9.96E-01
Phosphatidylinositol signaling system	1.54E-09	5.93E-01
Melanogenesis	2.02E-09	7.53E-01
Progesterone-mediated oocyte maturation	2.58E-09	8.07E-01
Type I diabetes mellitus	1.56E-08	1.24E-03
Vibrio cholerae infection	1.76E-08	9.96E-01
Asthma	4.50E-08	3.19E-03
Prion diseases	1.30E-07	2.41E-02
NOD-like receptor signaling pathway	1.46E-07	1.35E-01
Long-term depression	2.37E-07	9.96E-01
mTOR signaling pathway	3.31E-07	9.96E-01
Wnt signaling pathway	2.35E-06	1.83E-01
Aldosterone-regulated sodium reabsorption	2.75E-06	3.75E-01
Graft-versus-host disease	3.72E-06	6.57E-01
Vasopressin-regulated water reabsorption	1.95E-05	9.96E-01
Endocytosis	4.83E-05	2.71E-02
Oocyte meiosis	3.64E-04	7.25E-01
Dilated cardiomyopathy	6.46E-04	4.76E-01
Inositol phosphate metabolism	2.74E-03	9.96E-01
Thyroid cancer	3.59E-03	9.78E-01
Taste transduction	5.15E-03	9.96E-01
Neuroactive ligand-receptor interaction	1.40E-02	8.23E-01
Amyotrophic lateral sclerosis (ALS)	1.41E-02	9.96E-01
Olfactory transduction	1.44E-02	9.96E-01
Alzheimer's disease	1.49E-02	5.84E-01
