# Supplementary material for: Statistical Assessment of Crosstalk Enrichment between Gene Groups in Biological Networks
Source: PLoS One. 2013 Jan 23;8(1):e54945. doi: 10.1371/journal.pone.0054945 (PMC3553069; doi:10.1371/journal.pone.0054945)
Supplement: Text S1 — Pseudocode for randomization algorithm. (PDF) [file pone.0054945.s002.pdf]

Text S1: Pseudocode for randomization algorithm.

Link Permutation algorithm

The notation |*S*| is the cardinality (or size) of the set *S*.

Pseudo-code for Link Permutation

```
Begin
    Create vector linkSet and add all links from network to linkSet
    Create empty vector testedLinks
    while (|linkSet| >= 2  OR  |testedLinks| < |linkSet|):
        Pick a random link A-B from linkSet
        Pick a random link C-D from linkSet
        if (A-D and C-B not in network):
            Swap A-B and C-D to A-D and C-B
            Remove A-B and C-D from linkSet
        else if (A-C and B-D not in network)
            Swap A-B and C-D to A-C and B-D
            Remove A-B and C-D from linkSet
        else:
            Add link pair to testedLinks
    end while
End
```

Node Permutation algorithm

The *degNodesMap* is a map from an integer to a vector of nodes that have the same equivalence class established by the by the equivalence relation: $B[d] = \text{round}(\ln(d)+1)$, where *d* is each node's degree. A log scale is used so that scale free networks (such as observed in biological networks) will have approximately the same number of nodes in each bin. The notation *R.degree* is the degree of node *R* in the original network.

Pseudo-code for Node Permutation

```
Begin
    Create mapping of nodes from binned degree: degNodesMap
    Create set of nodes paired with degree in original network: recordSet
    Randomly shuffle recordSet
    for (R in recordSet):
        Pick a random node K from degNodesMap[B[R.degree]]
        Swap the labels of R and K
    end for
End
```

Link Assignment algorithm

Test condition for adding a link between *K* and *R*: *R* is not equal to *K*, link *R-K* is not already in the network, *R* AND *K* have not recovered their original degree yet. The notation *deg*(*K*) is the current degree of node *K* in the *network*. The notation *K.degree* is the degree of node *K* in the original network.

Pseudo-code for Link Assignment

```
Begin
    Create set of nodes paired with degree in original network: recordSet
    Randomly shuffle recordSet
    Remove all links from network
    for (k = 1 to |recordSet|):
        K = recordSet[k]
        Generate list of indices of recordSet: recordIndexSet
        while (deg(K) < K.degree OR  |recordIndexSet| == 0 OR |recordSet| == 0 ):
            Pick a random index from recordIndexSet
            R = recordSet[index]
            if (K and R can be linked in the network):
                Create R-K in network
                if (deg(K) ==  K.degree):
                    Remove K from recordSet
                    k = 1
                    break while
                if (deg(R) ==  R.degree):
                    Remove R from recordSet
                    k = 1
                    break while
            else:
                Remove index from recordIndexSet
        end while
    end for
End
```

Link Assignment + Second-order modification

The algorithm for link assignment + second order is similar to the link assignment algorithm, but instead of choosing a random node from the whole network (represented by *recordSet*) for node *K* to connect to, it picks from a set of nodes that fall into the same log degree bin [established by the equivalence relation: $B[d]$ = round(ln(d+1))], as the original neighbors of *K*. For example, say that node *K* was linked to a set of nodes that have the following degree sequence: {1, 30, 100, 400}. This can be termed the neighbor degree sequence of node *K*. By choosing nodes from the same *binned* neighbor degree sequence, the algorithm assures that node *K* has approximately the same neighbor degree sequence before and after randomization.

Fixing Degree Sequence Errors

For the two link assignment methods, a few nodes may not be able to conserve their degree due to conflicts in the test cases once the network becomes more densely connected. In that case the following procedure can be applied:

Pseudo-code for Fixing Degree Sequence Errors

```
Begin
    #Fix odd errors first
    Create a set of nodes that did not conserve degree: errNodes
    Sort errNodes in increasing order of deltaDeg(N) = N.degree - deg(N)
    for (N1 in errNodes):
        if (deltaDeg(N1) odd):
            N2 = next node in errNodes with deltaDeg(N2) odd
            for (link L1-L2 in network):
                if (testCase1):
                    Remove link L1-L2 from network
                    Create links (either form N1-L1 and N2-L2 or N1-L2 and N2-L1)
                    break for
            end for
    end for
    #Fix even errors next (errNodes should only have even errors at this point)
    for (N1 in errNodes):
        for (k=0 to deltaDeg(N1)/2):
            for (link L1-L2 in network):
                if (testCase2):
                    Remove link L1-L2 from network
                    Create links N1-L1 and N1-L2
            end for
        end for
    end for
End
```

The following are the test cases used above.
testCase1: the nodes *L1* OR *L2* have an odd degree, AND [the nodes *L1* AND *L2* are not nodes *N1* OR *N2*, AND [the network doesn't already have links *N1-L1* AND *N2-L2* OR links *N1-L2* AND *N2-L1*]].
testCase2: the nodes *L1* AND *L2* are not N1, AND the network doesn't already have links *N1-L1* AND *N1-L2*.
