# Supplementary material for: Statistical Assessment of Crosstalk Enrichment between Gene Groups in Biological Networks
Source: PLoS One. 2013 Jan 23;8(1):e54945. doi: 10.1371/journal.pone.0054945 (PMC3553069; doi:10.1371/journal.pone.0054945)
Supplement: Text S2 — Implementation Details. (PDF) [file pone.0054945.s003.pdf]

Text S2: Implementation Details.

CrossTalkZ requires a minimum of two input files, one specifying a network (network file) with nodes and links between nodes and another specifying a set of groups of nodes (group file). A group file is a tab-delimited list of gene/protein names in the first column and a respective group associated with it in the second column. An optional third column is a user specified field that can be used to describe the type of group, e.g. metabolic or signaling. The accepted formats of the network file are either an XGMML graph or a simple tab-delimited list of links between genes – columns 1 and 2 – and an optional column 3 for a weight (confidence, attribute etc.) of the link. Results from CrossTalkZ are written to a tab-delimited file and include the number of links expected by chance, the number of observed links, z-score, p-value, false discovery rate and reduced chi-squared value that indicates a deviation from normality of the distribution of the expected number of links.

Via the command-line interface, the user can specify several parameters: the number of iterations, a link weight cutoff, randomization method, the link counting mode, a custom result file, minimum genes allowed per group, and an option to simply write one randomized graph to a specified file. In general, more iterations of the randomization algorithm provide better statistics. If a link cutoff is specified, any link that has a weight lower than the cutoff is excluded, otherwise all links are included. There are two alternative methods to count links, one that doesn't count a link if *either* of the connected genes belongs to *both* groups (default) and the other will not count a link if *both* of the connected genes belong to *both* groups.
